# Modulation of Inflammation by Extracellular Granzyme A

**DOI:** 10.3389/fimmu.2020.00931

**Published:** 2020-05-19

**Authors:** Kim R. van Daalen, Josephine F. Reijneveld, Niels Bovenschen

**Affiliations:** ^1^Cardiovascular Epidemiology Unit, Department of Public Health and Primary Care, University of Cambridge, Cambridge, United Kingdom; ^2^Department of Pathology, University Medical Center Utrecht, Utrecht, Netherlands; ^3^Center for Translational Immunology, University Medical Center Utrecht, Utrecht, Netherlands

**Keywords:** Granzyme A, granzymes, inflammation, extracellular, inflammatory disease

## Abstract

Granzyme A (GrA) has long been recognized as one of the key players in the induction of cell death of neoplastic, foreign or infected cells after granule delivery by cytotoxic cells. While the cytotoxic potential of GrA is controversial in current literature, accumulating evidence now indicates roles for extracellular GrA in modulating inflammation and inflammatory diseases. This paper aims to explore the literature presenting current knowledge on GrA as an extracellular modulator of inflammation by summarizing (i) the presence and role of extracellular GrA in several inflammatory diseases, and (ii) the potential molecular mechanisms of extracellular GrA in augmenting inflammation.

## Introduction

Granzymes are a family of homologous serine proteases primarily expressed by a collective of cytotoxic cells, i.e., cytotoxic T lymphocytes (CTLs), γδ T cells, natural killer (NK) cells, NK-T cells. Classically the role of cytotoxic cells is described as promoting cytotoxic lymphocyte-mediated eradication of neoplastic, foreign or infected cells via the induction of (apoptotic) cell death (1). Apoptosis mediated by cytotoxic cells is induced via engagement of the death receptor pathway or the granule secretory pathway (2). Whereas the death receptor pathway involves death cell surface receptor-ligand interaction and caspase recruitment, the granule secretory pathway delivers granzymes through a process involving the aid of perforin, a pore-forming protein, to target cells (3, 4). Upon recognition of a target cell, cytotoxic cells release the content of granules into the immunological synapse. Perforin provides granzymes access to the cytosol of the targets cell, where granzymes cleave their cohort of substrates to promote programmed cell death (5, 6). To date, five human granzymes are known (granzymes A, B, H, K, and M), while ten mouse granzymes have been identified (7). Granzyme A (GrA, a tryptase) and granzyme B (GrB, an aspartase) being best characterized (3, 8). Although, human granzymes are highly homologous in amino acid sequence (40%), they variate in their primary substrate specificity (amino acid after which the granzyme preferably cleaves) resulting in a unique granzyme degradome (9).

GrA is the most abundant protease present in cytotoxic granules and is reported as dominant mediator of toxicity *in vitro* (4). Among serine proteases GrA has an unique quaternary structure consisting of a disulphide-linked homodimer of 60kDa linked via Cys93 (10). Dimerization creates a high degree of specificity for GrA due an extended site for its substrates (11). GrA cleaves substrate after Arg or Lys like its closest homolog GrK, whilst GrB cleaves after Asp or Glu, GrM after Leu or Met and GrH after Tyr or Phe (12). One intracellular inhibitor has been identified for GrA (Serpinb6b) (13), whilst two extracellular inhibitors have been reported [Kazal-type pancreatic secretory trypsin inhibitor (14), serpin antithrombin III (SERPINC1) (15)]. With its tryptase-like activity GrA has been shown to activate caspase independent cell death pathways with the cleavage of the mitochondrial protein NDUFS3 resulting in reactive oxygen species (ROS) generation. Triggered by ROS, the SET complex translocates to the nucleus where GrA cleaves SET complex components which result in opening of the DNA (due to targeting of histones) and single stranded DNA nicks (16–20). However, both human studies and *in vivo* mouse studies indicate that GrA by itself is not cytotoxic in contrast with initial *in vitro* reports (21). Furthermore, new *in vitro* studies also indicate contrasting results with native human GrA showing a lack of cytotoxicity *in vitro*, whilst recombinant mouse GrA studies contrastingly demonstrate cytotoxicity in several *in vitro* assays (4, 22). This may suggest alternatives roles for GrA.

**Figure 1:**
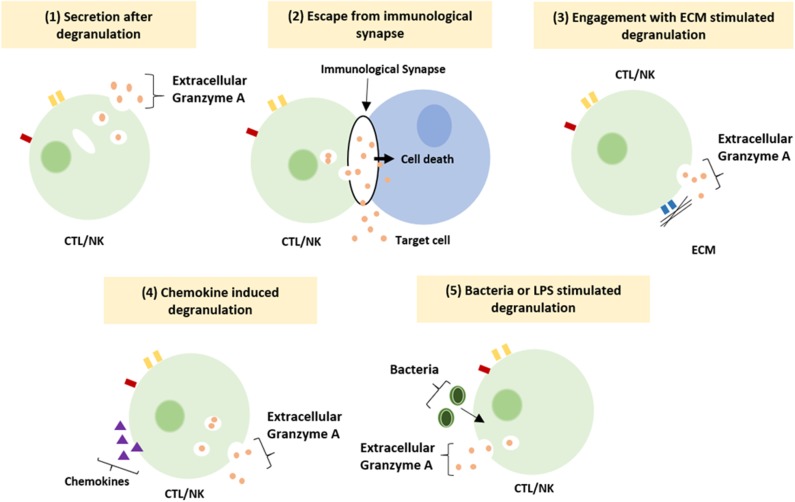
Suggested mechanism resulting in extracellular granzyme A release. Granzyme A can be release as a result of (1) constitutive granzyme A secretion after degranulation, (2) leakage of granzyme A from the immunological synapse, (3) degranulation after engagement of integrin with the ECM, (4) induction of degranulation by locally released chemokines (5) induction of degranulation by bacteria or LPS (8).

The functioning of GrB has recently been redefined by the discovery of multiple extracellular roles including the mediation of skin injury, inflammation and repair (23). Moreover, cumulative clinical and biochemical evidence indicate elevated levels of extracellular GrA in plasma, serum, synovial fluid and bronchoalveolar lavage (BAL) fluid. This includes patients with various viral infections, bacterial infections or other pro-inflammatory conditions (8, 24, 25). These elevated levels of extracellular GrA could potentially reflect spontaneous or inadvertent release of granzymes after elevated CTL/NK numbers in response to persistent inflammation, however extracellular biological effects are increasingly described (6, 8) (Figure 1). Furthermore, dendritic cells, mast cells and macrophages can express GrA but not perforin, suggesting perforin-independent (extracellular) roles for granzymes (26). Yet, little has so far been described on potential functioning of extracellular GrA.

This paper aims to explore the literature presenting current knowledge on GrA as an extracellular modulator of inflammation by summarizing (i) the presence and suggested roles of extracellular GrA in several inflammatory diseases, and (ii) the molecular mechanisms of GrA in augmenting inflammation.

## Extracellular Granzyme A During Inflammatory Disease

### Microbial Infections, Bacteria

A plethora of pathogenic micro-organism exists (bacteria, viruses, parasites, and fungi) that can infect the human body. Exposure typically leads to acute infection that with appropriate immune response subsides in the elimination of the involved pathogen within days (27). With 1.6 million deaths and 10 million cases in 2017 alone, tuberculosis (TB) remains a global health problem with impaired control by the emergence of drug resistant forms of *Mycobacterium tuberculosis* (28, 29). Extracellular plasma GrA levels are increased in patients with TB in comparison with control patients, suggesting that extracellular GrA could be a potential new therapeutic target in the inflammatory response to mycobacteria (29–31). In TB, extracellular GrA has been found to induce inflammatory responses that lead to intracellular anti-microbial activity *in vitro*. Co-culture of mycobacteria infected macrophages with either mycobacteria specific Vγ9Vδ2 T cells or purified GrA result in the production of the pro-inflammatory cytokines TNFα and IL-1β by macrophages. This alone or in combination with unidentified factors results in the inhibition of intracellular mycobacterial growth. The inhibitory mechanism is independent of perforin, apoptosis, autophagy, nitric oxide production, type I interferons, and Fas/FasL. Necessity of TNFα is clear, as anti-TNF neutralizing antibodies prevent the inhibitory activity of Vγ9Vδ2 T cells and TNF knockdown prevents inhibition of intracellular mycobacterial growth. As high levels of TNFα is a marker of uncontrolled mycobacterial growth, it is currently believed that *in vitro* GrA mediated inhibition of mycobacterial growth includes other factors in addition to TNFα. The complete molecular signature responsible for GrA-mediated response and how TNFα influences mycobacterial growth therefore remains unknown (31). In an attempt to find a potential source of increased GrA *in vivo*, correlations between intracellular and extracellular granzymes were analyzed in active TB patients. However, this did not yield any significant results for the human lymphocyte populations studied (30). Furthermore, a recent mouse study on the *in vivo* role of GzmA in tuberculosis infection contradicts *in vitro* results. Although, GzmA is expressed by cytotoxic cells from mouse lungs during infection, GzmA knockout mice show no difference in lung bacterial burden compared to wildtype mice in long and short-term *M. tuberculosis* infection. This suggests extracellular GrA may not have a protective role *in vivo* in tuberculosis response (32).

Studies on sepsis and experimental endotoxemia found extracellular GrA plasma levels significantly higher in severe sepsis, septic shock and endotoxemia (33, 34). Experimental endotoxemia, a well-accepted model of systemic inflammation in humans, was induced by intravenous injection of the endotoxin LPS in volunteers. Extracellular plasma GrA increases peaking at 2 h post administration and associates with a decrease in the number of CTL and NK cells in the circulation. The latter suggests that LPS administration results in the activation of cytotoxic lymphocytes *in vivo*, which results in a quick granzyme release (33). Patients with melioidoses caused by *Burkholderia pseudomallei*, a gram-negative bacterium, also indicate increased serum levels of GrA in comparison with control subjects on admission and remained high during the 72 h study period. Serum GrA levels were not significantly elevated in patients with non-bacteremic melioidosis (33). Additionally, while patients infected with *Salmonella enteric* (1) in typhoid fever and *Streptococcus Pneumoniae* (35) in community-acquired pneumonia had elevated GrA levels in acute phase plasma and BAL, respectively, patients with *Neisseria meningitides* (36) infection did not, except for patients with shock. Extracellular plasma GrA levels in patients with typhoid fever correlate with IFN-γ, which is critical in the systemic control of *S. enterica* infections (1, 35). Combining these findings from several bacteria with the observation that stimulation with endotoxin strongly induces secretion of extracellular GrA, GrA release is most likely a general (acute-phase) immune response during bacterial infection and not specific for certain species (33).

Experimental results in mice suggest that GrA-induced release of pro-inflammatory cytokines contribute to the development of sepsis during infection with *Brucella microti*, without being essential for clearance of *B. microti*. GrA^−/−^ mice injected with a sublethal dose of the bacterium clear the infection like wild-type (WT) mice, while GrB^−/−^ mice, perforin^−/−^ mice and mice depleted of cytotoxic CD8^+^ T cells do not. GrA^−/−^ mice have a higher survival rate compared with WT mice and perforin or GrB depleted mice, which is correlated with a significant reduction in the levels of the cytokines IL-1α, IL-1β, IL-6. Transfer of WT NK cells into GrA^−/−^ mice reverses susceptibility to sepsis, indicating that NK cells are the source of GrA during the infection (37). As both perforin^−/−^ and perfxgzmAxB^−/−^ mice are equally susceptible to sepsis as WT mice, this study was unable to determine whether the participation of GrA occurs intracellularly or extracellularly (7, 37). In agreement with this, similar studies reported that GrA^−/−^ mice are more resistant to LPS induced toxicity compared to WT mice, GrA^−/−^ mice show increased survival and lower bacterial load in BAL during *S. Pneumoniae* infection and GrA^−/−^ mice have reduced levels of pro-inflammatory cytokines during *S. Pneumoniae* infection (21, 35, 38). These data support a role for this granzyme in pro-inflammatory cytokine signaling during infection. By targeting GrA, bacteria-mediated sepsis can therefore be ameliorated without compromising the ability of the immune system to control infection. Exact molecular mechanisms are to be determined (7).

### Microbial Infections, Viruses

In addition to bacterial infections, viruses can infiltrate the human body and initiate an immune response mediated by cytotoxic lymphocytes (39). Longitudinal plasma samples from EBV-infected patients and one HIV-1 infected patient show increased levels of GrA in plasma during the acute phase of the infection and subsequent decline during its resolution. Increase in plasma GrA levels occur simultaneously with early markers of infection (40). Similarly, elevated plasma GrA is found in patients with dengue fever and cytomegalovirus infection after renal transplantation and in patients with respiratory syncytial virus infection (41–43). In the latter infection, plasma GrA clearly correlates with increased IL-8 levels and white blood cell counts after acute onset respiratory tract illness (43). In both non-human primates (44) and in chikungunya fever patients (44, 45), circulating GrA levels are elevated after chikungunya virus infection. Peak levels coincide with peaks of circulating IFNγ levels, increased viral load and disease scores in these patients (44, 45). It may seem conceivable that GrA contributes to the clearance of viral infections in humans by inducing a pro-inflammatory immune response in accordance with the correlated upregulation of cytokines and extracellular GrA in viral infections. Cytokines could directly inactivate the intracellular virus or they recruit and activate immune cells to remove infected cells (4). Yet, *in vivo* conflicting knockout mouse studies suggest these hypotheses may not be reliable. GrA deficiency does not seem to affect susceptibility to infections with e.g., chikungunya virus (44) and lymphocytic choriomeningitis virus (LMCV) (46). In contrast, GrA deficiency exhibited increased susceptibility to mousepox ectromelia (Ect) (47), herpes simplex virus (HSV) (48) and mouse cytomegalovirus (MCMV) (49). Control of the latter pathogens is often only delayed, without compromising mice survival, suggesting compensation mechanisms for absent GrA (50). Subcutaneous injection of enzymically active recombinant mouse GrA was able to mediate inflammation of chikungunya, zika and dengue virus (45).

Moreover, it has been found that several granzymes [e.g., GrH (51, 52), GrB (52), GrM (53)] can cleave viral [e.g., adenoviral DNA-binding protein (DBP) (52), phosphoprotein 71 (pp71) (53)] and host cell proteins involved in protein synthesis [e.g., La (51)] leading to restriction of viral replication (9, 54). In addition, the SET complex (a GrA/GrK substrate) has shown to be required for efficient HIV-1 infection by preventing autointegration (55) and for transcription of early genes during adenovirus infection (56). Whether extracellular GrA might also be able to cleave viral surface proteins, viral proteins expressed on the surface of infected cells, or host-surface proteins involved in the viral infection needs to be further explored (5, 9, 54). Interestingly, several viruses [e.g., cowpox virus (57), myxoma virus (58)] have been reported to encode proteins that inhibit GrB, suggesting immune evasive adaptations (59, 60), whilst no viral inhibitors have been described for GrA in the current literature.

### Microbial Infections, Parasites

Parasites comprise diverse organisms with complex life cycles and often life-cycle specific interactions with the host immune systems. Many are long-term (chronic) persistent in the host due inadequate host immunity. Limited studies explored extracellular GrA in parasitic infections. A study in Cameroon showed that children presenting with clinical malaria (*Plasmodium falciparum*) have significantly increased concentrations of extracellular plasma GrA. Likewise, five healthy Dutch volunteers with no prior exposure to malaria that were experimentally infected by *P. falciparum* displayed extracellular plasma GrA increment 1-2 days prior to clinical symptoms and microscopically detectable parasitemia. This coincided with increases in IFNγ, IL-12p40, and IL-8. Although, granzymes are often considered as marker for CTL/NK involvement, additional roles in inflammation cannot be excluded and have to be determined (61). A study exploring the *in vivo* role of GzmA in mice showed that GzmA deficiency did not impact the outcome of *Leishmania major* infection (62). Other *in vivo* mice studies [e.g., on *Trypanosoma cruzei* (63)] used double knock-outs (e.g., GrA × B^−/−^) and therefore precludes drawing conclusions on GrA.

### Rheumatoid Arthritis

Rheumatoid Arthritis (RA) is a chronic autoimmune disease characterized by inflammation of the joints (synovitis), autoantibodies, systemic inflammation and cartilage and bone destruction (64, 65). The synovial membrane is infiltrated by multiple immune cells, including T cells, B cells, macrophages, and NK cells. Inflammation, predominantly mediated by IL-1 and TNF, leads to joint swelling and pain (64, 66). The final outcome of the disease is destruction of the joint (64, 65). Although external factors regulating TNF are well-known, endogenous factors may amplify TNF expression.

Increased levels of GrA in plasma, synovial fluid, and synovial tissue have been described in RA patients (40, 41, 66, 67). Markedly higher levels of GrA have been recorded in synovial fluid (up to 10-fold higher than patients with osteoarthritis or reactive arthritis), whereas GrA plasma levels are more similar to those of healthy controls. This suggest a local GrA release (40, 66). Mice with collagen induced arthritis (CIA) have significantly elevated extracellular levels of GrA in their joints and plasma and activated CLs at early and late RA stages. CIA was only slightly reduced in perforin^−/−^ mice, therefore the function of GrA seems perforin independent (68). Together with the finding of activated cytolytic cells in RA synovium, these results strongly suggest that GrA is synthesized and secreted locally in the rheumatoid joint and plays a role in the pathogenesis of RA (24, 33). However, further studies are required to validate and explore these hypotheses.

The function of extracellular GrA in synovial inflammation and joint destruction remains unclear. As for bacterial sepsis, GrA promotes inflammation of the joint by stimulating the release of pro-inflammatory cytokines (7, 37, 66). IL-1β, TNF-α, IL-6, and IL-8, produced mainly by macrophages and fibroblasts, are abundantly present in the synovium of RA patients (66). CIA mice have increased levels of IL-6 and TNF-α during both early and late stages of RA compared with WT mice, whereas these cytokines are reduced in GrA^−/−^ CI mice (68). Furthermore, GrA may contribute to rheumatoid arthritis partly by promoting mice osteoclast precursor differentiation via the stimulation of TNF-α secretion of monocytes and osteoclast precursors present in the inflammatory joint (68). Osteoclasts are cells that carry out bone resorption and have been shown to contribute to joint destruction in RA (69). Finally, ECM degradation induced by extracellular GrA might contribute to pathogenesis. Biologically active fibronectin fragments found in the synovium can induce neutrophil and monocyte chemotaxis, induce matrix metalloproteinase (MMP), induce chondrocyte aggrecanase expression and disrupt chondrocyte cell adhesion (8). ECM degradation may assist migration of activated cytotoxic T lymphocytes through the endothelial basement membrane and facilitate the influx of mononuclear cells contributing to hyperplasia and joint destruction (70).

### Inflammatory Lung Disease

Numerous lung diseases are characterized by the presence of activated alveolar CTL and NK cells. Active GrA levels are locally increased in the BAL fluid, but not in blood, of patients with CD8^+^ T-cell-mediated hypersensitivity pneumonitis (HP) compared to control subjects. This is in contrast with increased plasmatic GrA concentrations in several viral, bacterial and parasitic infections (Table 1). Not surprisingly, TNFα, IL-6, and IL-8 are increased in BAL of HP patients coinciding with GrA levels (71). GrA positive cells and increased levels of GrA in sputum are also found in some smoking patients with asthma, smokers and nonsmoking patients with asthma (73, 74, 78). Both support a role for extracellular GrA in the lung inflammatory response. In allergic asthma there was no increase of GrA in BAL consistent with the absence of a lymphoid source (e.g., CTLs) (73, 74, 78). Moreover, GrA mRNA and IL-1β, IL-6, IL-8, and TNFα mRNA were upregulated in cells from BAL of patients with acute respiratory distress syndrome following sepsis. Though indirect, it has been suggested that this might indicate elevated extracellular GrA levels (79).

**Table 1:** Detection of extracellular granzyme A in patients with infections or pro-inflammatory disease.

**Disease status**	**Extracellular space**			
	**Plasma/Serum**	**Synovial fluid**	**BAL**	**Sputum**
**Lung disease**				
Asthma Hypersensitivity pneumonitis (HP) Chronic obstructive pulmonary disease (COPD)	− (71) − (72)		− (73) ↑ (71)	↑ (74) ↑ (75)
**Viral infection**				
Chikungunya virus (CHIKV) Cytomegalovirus (CMV) Dengue virus (DENV) Epstein-barr virus (EB) Human immunodeficiency virus (HIV) Respiratory syncytial virus (RSV)	↑ (44, 45) ↑ (41, 42) ↑ (41) ↑ (40) ↑ (40) ↑ (43)			
**Bacterial infection**				
*Burkholderia pseudomallei* (melioidosis) *Mycobacterium tuberculosis* *Neisseria meningitidis*^a^ *Salmonella enterica* *Streptococcus Pneumoniae* Endotoxemia^b^ Severe sepsis^c^ Septic shock patients^c^	↑ (33) ↑ (29, 30) − (36) ↑ (1) ↑ (33) ↑ (34) (34) ↑ (35)			
**Parasitic infection**				
*Plasmodium falciparum*	↑ (61)			
**Arthritis**				
Rheumatoid arthritis (RA) Osteoarthritis (OA) Reactive arthritis	↑ (41, 66), (40) − (66) − (66)	↑ (40, 66, 67)		
**Other**				
Acute renal allograft rejection^d^ Behçet's disease Celiac disease Cow's milk protein sensitive enteropathy	− (42) ↑ (76) ↑ (77) ↑ (77)			

*BAL, bronchoalveolar lavage Symbols: ↑: higher levels compared to healthy individuals; −: no difference or non-significant difference*.

a *Only increased in patients in shock (although marginally)*.

b *Experimental human endotoxemia was used as a well-accepted model of systemic inflammation in humans. Volunteers received a bolus of intravenous injection of Escherichia coli endotoxin (LPS) (33)*.

c *Patients suffered from gram negative or positive infections, respectively. Some patients suffered from infection by multiple microorganisms. In some patient no infectious agent was found (34)*.

d *Concurrent viral infections were absent during acute rejection episode (42)*.

*Sole inclusion of primary research*.

Immunohistological studies indicate GrA expression by CTLs, NKs, alveolar macrophages, bronchiolar epithelium and type II pneumonocytes in both control subjects and chronic obstructive pulmonary disease (COPD) patients. GrA expression is significantly increased in sputum and/or lung specimens of patients with COPD in comparison with controls, but not in blood (72, 75, 80). Like in HP this suggest local lung GrA expression and tissue destruction (72). Several roles have been postulated in COPD. IL-6 and IL-8 show increased expression in COPD patients, indicating potential pro-inflammatory roles of GrA (80). Conjointly, recombinant rat GrA was found to cause rounding and detachment of an alveolar type II epithelial cell line (A549 cells), probably through its ability to cleave ECM, and was found to stimulate IL-8 via a mechanism involving microtubule disruption. This suggests that GrA might be involved in lung disease pathogenesis by loss of alveolar wall structures, chronic inflammation and neutrophil accumulation (81). It is noteworthy that the sulfated oligosaccharide k-carrageenan can inhibit the GrA promoted detachment of A549 cells *in vitro*, releasing IL-8. This might be of use for clinical purposes (82). Examination of BAL fluid and GrA^−/−^ mouse models could provide further insights in the role of GrA in COPD patients.

In conclusion, locally elevated extracellular GrA is likely to play a role in general lymphocytic lung disease by enhancing inflammation in the lungs through release of pro-inflammatory cytokines and contributing to tissue damage. Nevertheless, additional research is required on the multifactorial roles of GrA in lung tissue destruction.

### Other Inflammatory Diseases

Other diseases, such as Behçet's disease (BD), celiac disease (CD) and cow's milk protein sensitive enteropathy (CMSE), also show elevated GrA in serum and/or plasma (76, 77). Various micro-organisms, neutrophil hyperfunction, and autoimmune manifestation have been implicated as causative agents in BD. Using a BLT esterase assay, active BD patients show increased GrA levels in supernatants of lymphocytes correlated with the Vγ9Vδ2 expansion factor, suggesting active participation of CTLs and GrA in BD pathogenesis. The biological role of extracellular GrA in BD has not been studied. As GrA levels are elevated in both supernatant and serum, an extracellular role for GrA is speculated (76). Additionally, the intestinal immune response in both CMSE and CD is manifested by increased serum GrA, GrB and CD30. Elevated GrA correlates with the extent of CD villous atrophy. This offers new complementary means for diagnostic assessment of these diseases. More comprehensive studies on the detailed association and skewing of gut-associated lymphoid tissue and GrA functioning are needed (77).

### Tumors

Questions are raised about the impact of pharmacological/biological inhibition of GrA in inflammatory carcinoma. Considering the variety of roles of extracellular GrA in immune response described, it has been speculated that extracellular GrA may contribute to the cancer response and/or tumor promotion. For example, ulcerative colitis (UC) patients have higher levels of infiltrating lymphocytes expressing GrA (83) and respond better to anti-inflammatory immunotherapy correlating with decrease in GrA (84). However, such observations could be circumstantial and not related to extracellular GrA functioning. Currently, there is a lack of (published) human, animal and *in vitro* studies in this context so far. Efforts to properly understand the potential role of GrA (intracellular and extracellular) in tumor progression can hold great promise for therapeutic approaches.

## Pro-inflammatory Mechanisms of Extracellular Granzyme A

**Figure 2:**
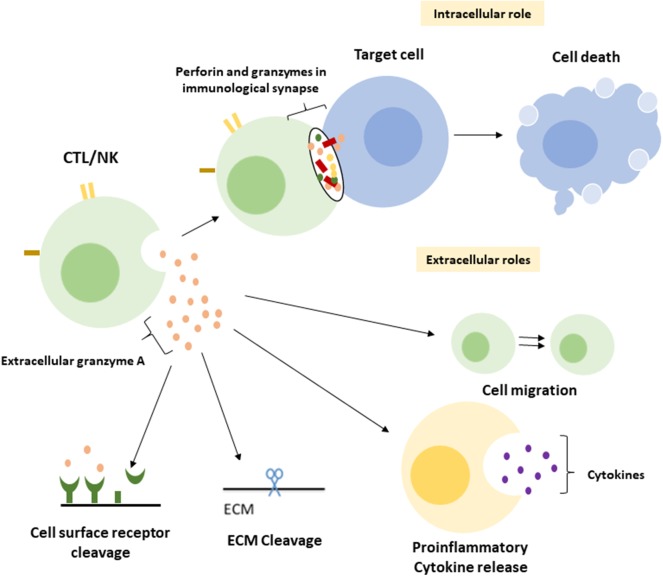
Intracellular and putative extracellular functions of Granzyme A. Classically GrA and other granzymes have been described as promoting cytotoxic lymphocyte mediated eradication of target cells via the induction of (apoptotic) cell death. Upon recognition of the target cell CTL release granule content into the immunological synapse, perforin provides access to the cytosol and granzymes promote cell death intracellularly (1). During a number of inflammatory disease statues GrA accumulates in extracellular space and is suggested to (i) induce release of pro-inflammatory cytokines in fibroblasts, epithelial cells, monocytes, and macrophages (3, 85, 86), (ii) remodel extracellular matrix (87–91), (iii) contribute to the migration of activated CTLs through tissue and extravasation of these cells from the vasculature (88), and (iv) cleavage of (cell surface) receptors as the Thrombin like receptor in neurite retraction (92–94).

A central function of GrA in the modulation of pro-inflammatory cytokine expression, which is at least partly enhanced upon GrA intracellular delivery, is postulated (Figure 2) (95). Granzymes are localized both intra-and extracellular and thereby possess the potential to cleave on both sides of the membrane (96). Extracellular substrates for GrA are beginning to emerge. While direct *in vivo* evidence of granzyme-mediated cleavage of extracellular substrates is limited under physiological conditions, several substrates *in vitro* (Table 2) have formed the basis for hypothesis formulation linking multifactorial extracellular GrA activity to disease pathogenesis (Table 1).

**Table 2:** Extracellular substrates of GrA and suggested biological impact.

**Substrate**	**Suggested biological impact**	**References**
Basement membrane proteoglycans	Liberation basic fibroblast growth factor, protection against inhibition by natural high molecular weight inhibitors, lymphocyte migration.	(87)
Collagen IV	Influence on lymphocyte migration, anoikis, cell adhesion. Reduction adhesion of epithelial cells with cell-basement membrane.	(88, 97)
Fibronectin	Influence on lymphocyte migration (through fibrin clots), anoikis, cell adhesion. Reduction adhesion of epithelial cells with cell-basement membrane.	(89, 97)
Myelin basic protein (MBP)	MBP degradation resulting in myelin destruction. Pathogenesis multiple sclerosis.	(98)
Pro-urokinase plasminogen activator	Convert single-chain human pro-urokinase into active two-chain enzyme Roles in plasmin generation	(99)
Thrombin-like receptor on neurites Platelet thrombin receptor	Neurite retraction, reversed stellation of astrocytes Desensitized response to thrombin-induced aggregation by platelets	(92) (93, 94)
Unidentified (likely) cell surface receptor^a^	Pro-inflammatory cytokine production by fibroblasts, epithelial cells, monocytes, and macrophages.	(21, 31, 85, 86, 95)
*Proteinase-activated receptor 2 (PAR-2)^*b*^*	*Protease-activated receptor-2 activating peptide (SLIGRL) is yielded. Roles in promoting inflammation*.	(100)

a *Sower et al. found a 5-fold difference in potency between thrombin and GrA suggesting that granule-associated proteases may signal through other membrane proteins than the thrombin receptor. However, no such receptor has been identified yet. Release of pro-inflammatory cytokines is suggested to be on their own or potentiating LPS-induced responses (21, 31, 85, 86, 95)*.

b *Hansen et al. found that treatment of P20 peptide (corresponding to the cleavage/activation site of the wt-r PAR-2 N terminus) with GrA for 20 h yielded 22 ± 2% (n = 3) conversion to the PAR-2 -activating peptide. However, calcium mobilization experiments did not show activation of PAR-2 by GrA (data not shown in paper) (100) and another study notes that the tryptase fails to induce Ca influx to efficiently cleave various PAR sub-types (data not shown in paper) (21)*.

*As most studies have been performed in vitro or in mouse/rat models it is unclear to what extent these findings have physiological relevance in humans*.

### Cytokine Release and Activation

First evidence suggesting a role of GrA as pro-inflammatory mediator in cytokine activity was proposed almost 25 years ago when it was found that GrA can function *in vitro* as an IL-1β-converting enzyme. GrA, but not GrB, cleaves the 31 kDa precursor form (pIL-1β) into the active 17 kDa cytokine in cultured macrophages expressing pIL-1β (101). Physiological relevance of this has never been shown (3). Although some have been able to show GrA alone can convert IL-1β to its mature form via direct cleavage, others were unsuccessful reproducing this maturation or suggest the aid of the inflammasome (3, 21, 85, 102).

*In vitro* human extracellular GrA stimulates release of various pro-inflammatory cytokines in multiple cell-types; IL-6 from fibroblast cell lines (lung, intestinal) and IL-8 from both fibroblasts (lung, skin, intestinal) and cultured (lung) epithelial cell lines (86). Additionally, GrA stimulates the production of IL-6, IL-8 and TNF-α in human peripheral blood mononuclear cells and purified monocytes without the presence of perforin (103), whilst inducing macrophages to produce TNF-α and IL-1 β (21, 85). Recently it has been shown that, although able to enhance the cytokine response induced by LPS, extracellular GrA is not enough to induce release of cytokines from human monocytes (26). At the same time, depletion of caspase-1 almost completely inhibits the release of IL-1β and IL-6 in monocytes, supporting arguments for involvement of an inflammasome. In contrast, GrA internalized by human blood derived-macrophages GrA can independently enhance cytokine expression (3, 102). A new model proposed that GrA in murine macrophages is induced by bacterial toxin, via the JAK-STAT pathway. GrA is subsequently released extracellularly via exocytosis and taken up by other macrophages, where it directly induces conversion of intracellular pro-IL-1β into mature IL-1β (102).

The molecular mechanism by which granzymes directly release pro-inflammatory cytokines remains unclear. Catalytic activity seems to be required for cytokine release and internalization of GrA partly enhances its release, suggesting activation of down-side effectors localized both internally and externally of cytokine producing cells (3, 21, 85, 86). Recently, we have examined the role of GrA in potentiating toll-like receptor (TLR) mediated cytokine response. Extracellular GrA potentiates a marked increase in TLR2/4 agonist-induced pro-inflammatory cytokines. Interestingly, inactive mutant GrA (Ser to Ala substitution in the GrA catalytic center) results in similar cytokine response as WT GrA, indicating an independence of the GrA catalytic activity. Thus, GrA can use its proteolytic activity to release cytokines in the absence of TLR stimulation or inactive GrA can make use of TLRs (95). Multiple studies suggest the presence of unidentified GrA-sensitive receptors on the cell-surface of reacting cells (21, 31, 85, 86, 95, 96).

As granzyme activity is tightly regulated by serine protease inhibitors *in vivo* and extracellular GrA in complex with proteoglycans is resistant to serpins, regulation of granzyme activity might be essential to fine-tune pro-inflammatory cytokine response (95). GrA-inducible cytokines have been linked to diseases beyond those described above suggesting alternative roles to the traditionally proposed cytotoxic role for GrA in chronic inflammation (96).

### Extracellular Matrix Remodeling

Early evidence of extracellular GrA disrupting basement membrane proteins was proposed in a study that showed that high GrA concentrations *in vitro* could cleave the α2(IV) chain of collagen IV in mice (88). Additionally, extracellular GrA cleaves fibronectin, myelin basic protein and heparan sulfate proteoglycans *in vitro*, suggesting a role in ECM remodeling (87–91, 97). Destruction and remodeling of the ECM could contribute to the migration of activated cytotoxic T lymphocytes through tissue and extravasation of these cells from the vasculature and to the pathogenesis of several viral, bacterial and parasitic diseases (88, 104). As GrA is elevated in COPD and identified extracellular GrA substrates are components of the alveolar ECM, GrA may contribute extracellularly to alveolar wall damage in COPD (75). Detachment of cells, such as long epithelial cells and small intestinal epithelial cells, due cleavage of ECM proteins, could lead to both cell death resulting in COPD in lung epithelial cells and maturation and exfoliation of cells in the intestine (81, 97). Furthermore, integrin interactions with ECM proteins (e.g., fibronectin and vitronectin) may induce T-cell activation and degranulation of GrA (8). Additional work is needed to establish the *in vivo* relevance of these findings.

### Receptor Cleavage

Several receptors are identified as external substrates of GrA, including e.g., thrombin-like receptor on neurites and platelet thrombin receptors. Cleaving of these receptors by GrA may activate them and/or induce inflammation in disease (92, 93, 100). By cleavage and subsequent activation of the thrombin receptor on neurites, immediate neurite retraction and reversed stellation of astrocytes is induced. This may contribute to the etiology of autoimmune disorders of the nervous system (92). In contrast, GrA cleavage of the platelet thrombin receptor desensitizes response to thrombin-induced aggregation by platelets. This might be a result of competition of GrA with thrombin for ThR binding (93, 94). PAR-2 has also been suggested as a substrate of GrA, although available data is limited and conflicting. PAR-2 is a G-protein coupled receptor activated through the cleavage of the receptor's extracellular N-terminal domain. This yields the PAR-2 activating sequence that can subsequently induce the expression of e.g. pro-inflammatory cytokines (96, 100). One study found that treatment of P20 peptide (corresponding to the cleavage/activation site of the wt-rPAR-2 N terminus) with GrA yielded conversion to the PAR-2-activating peptide. However, calcium mobilization experiments did not show activation of PAR-2 by GrA (100) and another study notes that GrA fails to induce Ca influx to efficiently cleave various PAR-2 sub-types (21).

## Concluding Remarks

Extracellular GrA is involved in several inflammatory diseases. After amplified debate on GrA's cytotoxic potential, novel functions challenge the traditional dogma of a protective role of GrA in immune homeostasis. The observations suggest that GrA might induce increased inflammation, (over)reaction of the immune system and pathogenesis (e.g., alveolar wall damage in COPD, sepsis, tumor promotion, rheumatoid arthritis). The elevated GrA levels in bodily fluids from patients with the described diseases and infections possibly arise from local release of GrA in the inflamed tissue. Considering its pro-inflammatory role, GrA is a potential target for anti-inflammatory interventions. Nevertheless, although implicated in cytokine release, ECM matrix remodeling and receptor cleavage, functional and pathological consequences of extracellular GrA release remain largely unknown. Further research is required to assess (i) mechanism and regulation of extracellular GrA release, (ii) extracellular and intracellular pathways activated for the promotion and/or release of cytokines, (iii) direct involvement of GrA in disease pathogenesis (e.g., virus infections, parasitic infections, cancer promotion, (iv) potential usage of GrA as target in therapies without ameliorating the immune systems' ability to control infections, e.g. by using the native GrA inhibitors, (v) the potential to use extracellular GrA expression for diagnostic tools, and (vi) whether known GrA inhibitors in the extracellular matrix (or other mechanism) are altered in favor of increased GrA activity in inflammatory disease.

## Author Contributions

All authors listed have made a substantial, direct and intellectual contribution to the work, and approved it for publication.

## Conflict of Interest

The authors declare that the research was conducted in the absence of any commercial or financial relationships that could be construed as a potential conflict of interest.
